# Lipocalin-2 is associated with FGF23 in WNT1 and PLS3 osteoporosis

**DOI:** 10.3389/fendo.2022.954730

**Published:** 2022-09-08

**Authors:** Petra Loid, Helena Hauta-alus, Outi Mäkitie, Per Magnusson, Riikka E. Mäkitie

**Affiliations:** ^1^ Folkhälsan Research Center, Genetics Research Program, Helsinki, Finland; ^2^ Children’s Hospital, University of Helsinki and Helsinki University Hospital, Helsinki, Finland; ^3^ University of Helsinki, Helsinki, Finland; ^4^ Population Health Unit, Finnish Institute for Health and Welfare (THL), Helsinki, Finland; ^5^ Research Unit for Pediatrics, Pediatric Neurology, Pediatric Surgery, Child Psychiatry, Dermatology, Clinical Genetics, Obstetrics and Gynecology, Otorhinolaryngology and Ophthalmology, Medical Research Center Oulu, Oulu University Hospital and University of Oulu, Oulu, Finland; ^6^ Department of Molecular Medicine and Surgery, Karolinska Institutet, and Clinical Genetics, Karolinska University Hospital, Stockholm, Sweden; ^7^ Department of Clinical Chemistry, and Department of Biomedical and Clinical Sciences, Linköping University, Linköping, Sweden; ^8^ Department of Otorhinolaryngology–Head and Neck Surgery, Helsinki University Hospital and University of Helsinki, Helsinki, Finland

**Keywords:** lipocalin-2, monogenic osteoporosis, bone biomarkers, FGF23, PLS3, WNT1

## Abstract

**Background:**

The pathogenic mechanisms of early-onset osteoporosis caused by *WNT1* and *PLS3* mutations are incompletely understood and diagnostic biomarkers of these disorders are limited. Recently, lipocalin-2 has been recognized as an osteokine involved in bone development and homeostasis. However, the role of lipocalin-2 in WNT1 and PLS3 osteoporosis is unknown.

**Objective:**

We aimed to investigate if plasma lipocalin-2 could be utilized as a biomarker for WNT1 and PLS3 osteoporosis and to evaluate the association between lipocalin-2 and other parameters of bone metabolism.

**Methods:**

We measured plasma lipocalin-2 in 17 *WNT1* and 14 *PLS3* mutation-positive patients and compared them to those of 34 mutation-negative (MN) healthy subjects. We investigated possible associations between lipocalin-2 and several bone biomarkers including collagen type I cross-linked C-telopeptide (CTX), alkaline phosphatase (ALP), type I procollagen intact N-terminal propeptide (PINP), intact and C-terminal fibroblast growth factor 23 (FGF23), dickkopf-1 (DKK1) and sclerostin as well as parameters of iron metabolism (iron, transferrin, transferrin saturation, soluble transferrin receptor and ferritin).

**Results:**

We found no differences in plasma lipocalin-2 levels in WNT1 or PLS3 patients compared with MN subjects. However, lipocalin-2 was associated with C-terminal FGF23 in WNT1 patients (*r*=0.62; *p=*0.008) and PLS3 patients (r=0.63, p=0.017), and with intact FGF23 in PLS3 patients (*r*=0.80; *p*<0.001). In addition, lipocalin-2 correlated with serum transferrin in WNT1 patients (*r*=0.72; *p*=0.001).

**Conclusion:**

We conclude that plasma lipocalin-2 is not altered in *WNT1* or *PLS3* mutation-positive subjects but is associated with FGF23 in abnormal WNT1 or PLS3 signaling and with iron status in abnormal WNT1 signaling.

## Introduction

WNT1 and PLS3 osteoporosis are two monogenic forms of severe, childhood-onset osteoporosis characterized by low bone mineral density (BMD) and multiple peripheral and vertebral compression fractures, resulting in exaggerated kyphosis and loss of adult height (1–4). While the autosomal dominant WNT1 osteoporosis affects both sexes similarly, the X-linked PLS3 presents predominantly in males while females portray a variable phenotype from normal BMD to skeletal fragility with fractures. Bone biopsies show destruction in bone tissue microarchitecture, low number of bone cells and low bone turnover (1–4). Despite the severe and evident skeletal pathology, conventional metabolic bone markers are normal in affected individuals (1–3, 5, 6). We have previously surveyed novel bone markers in cohorts of 17 *WNT1* and 14 *PLS3* mutation-positive subjects and established that fibroblast growth factor 23 (FGF23) levels are significantly increased in WNT1 patients and dickkopf WNT signaling pathway inhibitor 1 (DKK1) levels in PLS3 patients (7). DKK1 and FGF23 are two osteocyte-derived hormones with key functions in bone metabolism.

Furthermore, WNT1 patients have been found to have low bone marrow iron storages without evident anemia (8), supporting the crucial role of WNT signaling in bone marrow hematopoiesis and link to myeloproliferative disorders (9, 10). While these studies have provided novel information on the pathomechanisms behind these diseases, the exact molecular details remain unknown. In addition, the role of iron metabolism in PLS3-related osteoporosis is not known.

Lipocalin-2, also known as neutrophil gelatinase-associated lipocalin, is a ubiquitously expressed protein involved in various physiological functions including innate immunity, metabolic homeostasis, apoptosis, cell differentiation and transport of fatty acids and iron (5, 11, 12). Recently, lipocalin-2 has been identified as a novel osteokine with high expression in bone (13). Lipocalin-2 levels are shown to predict future fracture risk and correlate with bone markers in healthy individuals. One of these factors is DKK1, previously reported to correlate with lipocalin-2 (14–16). Lipocalin-2 has been shown to be upregulated in humans and in animal models in response to reduced mechanical forces (17). Furthermore, mouse studies have shown that overexpression of lipocalin-2 impairs the differentiation of osteoblast and increases osteoclastogenesis, ultimately leading to skeletal fragility (18). On the other hand, global ablation of lipocalin-2 in mice induces osteopenia, likely due to altered energy metabolism that causes impairment of osteoblast differentiation and activity (19) While these studies suggest a key role for lipocalin-2 in bone metabolism, the exact molecular mechanisms and impacts on bone cell functions remain unknown.

To our knowledge, there is no previous data on the role of lipocalin-2 in monogenic forms of osteoporosis or in relation to aberrant WNT1 or PLS3 signaling in bone tissue. Given the reported link to DKK1 and its significance in bone metabolism, we sought to evaluate the significance and utility of lipocalin-2 as a novel biomarker in WNT1 and PLS3 osteoporosis and its correlations with other parameters of bone turnover as well as with parameters of iron metabolism. We report unaltered lipocalin-2 levels in *WNT1* and *PLS3* mutation-positive subjects but a strong association with FGF23 concentrations. We also observed a correlation with parameters of iron status in patients with *WNT1* mutation.

## Methods

### Study subjects

The study cohort comprises *WNT1* and *PLS3* mutation-positive subjects from five unrelated Finnish families. The subjects were recruited through an ongoing research project on monogenic osteoporosis at the Helsinki University Hospital, as previously described (1, 3, 6, 7). Altogether 17 *WNT1* mutation-positive subjects consented to participate; they were all previously determined to harbor the same heterozygous *WNT1* missense mutation c.652T>G (p.C218G) resulting in early-onset osteoporosis; none of the patients had biallelic *WNT1* variants. For the *PLS3* patients, altogether 14 consented. They harbored different mutations, all resulting in early-onset osteoporosis: nine with an intronic splice site mutation c.73–24T>A (p.Asp25Alafs*17), three with an intragenic tandem duplication within *PLS3*, and two with a *PLS3* nonsense mutation c.766C>T (p.Arg256*). Apart from skeletal fragility, all mutation-positive subjects lacked clinical features of OI such as blue sclerae, dentinogenesis imperfecta, bone deformities or cardiac manifestations. To form a comparable control group, we also offered participation to all mutation-negative family members and subsequently 34 of them consented. All subjects signed a written informed consent upon participation. All genetic and clinical studies were approved by the Research Ethics Board of Helsinki University Hospital.

### Biochemical evaluation

Blood samples were drawn in the morning between 8 and 9 a.m. after an overnight fast. Serum and plasma aliquots were immediately stored at –80°C until analyses. Plasma lipocalin-2 levels were measured using the Human NGAL ELISA (Cat# HK330-02, Hycult Biotech, Uden, The Netherlands). The detection level for lipocalin-2 was 0.4 ng/ml. Serum concentrations of ionized calcium, phosphate, and alkaline phosphatase (ALP) were analyzed by routine clinical laboratory assays at the HUSLAB Laboratory, Helsinki, Finland. Serum 1,25-dihydroxyvitamin D (1,25[OH]_2_D) was analyzed with a chemiluminescent immunoassay (CLIA) on a LIAISON XL analyzer (DiaSorin, Stillwater, MN, USA). Both parathyroid hormone (PTH) and collagen type I cross-linked C-telopeptide (CTX) were determined by CLIA assays on the IDS-iSYS automated immunoassay system (Immunodiagnostic Systems, Ltd., Bolton, UK). Serum type I procollagen intact N-terminal propeptide (PINP) was measured with the UniQ radioimmunoassay (Aidian Oy, Espoo, Finland). Serum intact FGF23 (iFGF23) was determined by an enzyme-linked immunosorbent assay (ELISA) (Cat# CY-4000, Kainos Laboratories, Inc., Tokyo, Japan), and C-terminal FGF23 (cFGF23) was also assessed by ELISA (Cat# BI-20702, Biomedica, Vienna, Austria). Both dickkopf-1 (DKK1) (Cat# BI-20413) and sclerostin (Cat# BI-20492) were assessed by ELISA (Biomedica). These biomarkers were applied as potential modifiers or covariates, and specific methodological information is reported elsewhere (7).

Parameters of iron status were analyzed by routine clinical chemistry assays on a Cobas instrument (Roche Diagnostics Scandinavia AB, Bromma, Sweden) at the Department of Clinical Chemistry (accreditated testing laboratory, Swedac no. 1342), Linköping University Hospital, Sweden, with total analytical CVs of ≤4%. In brief, serum iron was determined by the colorimetric FerroZine method without deproteinization; transferrin and soluble transferrin receptor by immunoturbidimetry; and ferritin by electrochemiluminescence immunoassay. Transferrin saturation (%) was calculated by the formula = serum iron (µmol/L) × 100/serum transferrin (g/L) × 25.

### Statistical analysis

We present the data as means and standard deviations (SD) or medians and interquartile ranges (IQR). Normality of variables was visually examined. For non-normally distributed variables logarithmic transformation was conducted where applicable. We applied Pearson Chi-Square test to examine differences in iron, ionized calcium and 1,25(OH)2D status prevalences between subgroups of WNT1, PLS3 and MN subjects (**Table 1**). Independent-Samples Kruskal-Wallis test was conducted to study mean differences in iron and calcium metabolism markers without normal distribution, and ANOVA with variables of normal distribution between subgroups (**Table 1**). Independent-Samples T-test was applied to examine mean differences in lipocalin-2 and iron status markers of MP and MN subjects, and ANOVA among subgroups of WNT1, PLS3 and MN subjects with log-transformation as needed (**Table 2**). ANCOVA was used in adjusted models (**Table 2**). Bonferroni correction was applied as applicable. Covariates were chosen based on consistent correlations with dependents (lipocalin-2 and iron status indicators) and literature. Final model of ANCOVA was adjusted for iFGF23, cFGF23, PTH and age (**Table 2**). We reported Pearson correlation coefficients between lipocalin-2 and other biomarkers in entire population and in subgroups of WNT1, PLS3 and MN subjects. Interactions were tested for gender by stratification and as an interaction term. We identified possible outliers by visually examining Q-Q-plots and variable distributions and detecting extreme values with SPSS diagnostics (in lipocalin-2 four outliers were identified). Then we excluded the outliers and repeated the corresponding analyses to evaluate validity of our analyses. Results did not change significantly from original analyses confirming robustness of our findings. A *p* value of <0.05 was considered statistically significant. We used IBM SPSS Statistics 28 (IBM, NY).

**Table 1:** Subject characteristics and prevalence of normal iron status markers in *WNT1* and *PLS3* mutation-positive subjects compared with mutation-negative controls (MN).

Parameter	WNT1	PLS3	MN	*p* value
Cohort demographics
n	17	14	34	
Female, % (n)	77 (12)	64 (9)	50 (17)	0.18
Age, y, median [range]	52 [11-76]	41 [8-76]	36 [8-77]	0.39
Under 18 y, % (n)	24 (4)	29 (4)	21 (7)	0.84
Clinical characteristics^1^				
Prior bisphosphonate treatment, % (n)	41 (7)	43 (6)	6 (2)	
Ongoing bisphosphonate treatment, % (n)	12 (2)	7 (1)	0 (0)	
>2 peripheral fractures, % (n)	41 (7)	29 (4)	21 (7)	
Vertebral compression fractures, % (n)	47 (8)	64 (9)	9 (3)	
Abnormal renal function, % (n)	0 (0)	0 (0)	0 (0)	
Analyzed parameters				
Ferritin within reference range for age, % (n)	88 (15)	77 (10)	66 (21)	0.11
Iron within reference range for age, % (n)	94 (16)	69 (9)	94 (30)	–
Soluble transferrin receptor within reference range for age, % (n)	88 (15)	92 (12)	78 (25)	–
Transferrin within reference range for age, % (n)	100 (17)	92 (12)	81 (26)	–
Transferrin saturation within reference range for age, % (n)	94 (16)	69 (9)	78 (25)	0.60
Hemoglobin within reference range for age, % (n)	94 (16)	100 (14)	97 (32)	–
1,25[OH]_2_D within reference range for age, % (n)	94 (16)	93 (13)	92 (31)	–
Mean (SD) 1,25[OH]_2_D, pmol/L	114 (36)	150 (37)	121 (46)	**0.046^2^**
Ionized calcium within reference range for age, % (n)	82 (14)	93 (13)	97 (33)	–
Mean (SD) ionized calcium, mmol/L	1.26 (0.06)	1.23 (0.05)	1.25 (0.04)	0.27

Significance tested with Pearson Chi-Square, Independent-Samples Kruskal-Wallis and ANOVA if number of subjects adequate. Prevalence tested between both WNT1 and PLS3 mutation-positive subjects compared with mutation-negative subjects (MN). One value missing from hemoglobin, and three values from iron parameters. Reference ranges for age (females/males) (as according to HUSLAB, Fimlab and Nordlab): Ferritin F/M 6–17 yrs 6–70 µg/l 6–320 µg/l, F/M ≥18 yrs 13–150 µg/l/30–400 µg/l; Iron F/M 14–17 yrs 7–32 µmol/l/10–31µmol/l, F/M ≥18 yrs 9–34 µmol/l; soluble transferrin receptor F/M 7–12 yrs 2.0–5.1 mg/l/2.4–5.7 mg/l, F/M 13–17 yrs 1.6–5.2 mg/l/2.0–6.8 mg/l, F/M ≥18 yrs 1.8–4.6 mg/l/1.8–4.7 mg/l; transferrin <13 yrs 2–3.5g/l, >13 yrs 1.75–3.13 g/l; transferrin saturation <13 yrs 12–43%, >13 yrs 17–52%; hemoglobin M 8–11 yrs 116–154 g/l, M 12–13 yrs 124–161 g/l, M 14–15 yrs 130–170 g/l, F 12–15 yrs 120–154 g/l, F/M >16 yrs 117–155 g/l/134–167 g/l, 1,25-dihydroxyvitamin D (1,25[OH]_2_D) 48–190 pmol/L; ionized calcium 1.16–1.30 mmol/L.

^1^Clinical data as previously reported in (7).

^2^Pairwise comparison with Bonferroni correction showed no significant difference between groups (p>0.06). P values <0.05 highlighted in bold.

**Table 2:** Lipocalin-2 and indicators of iron status in *WNT1* and *PLS3* mutation-positive subjects compared with mutation-negative (MN) family controls.

	WNT1	PLS3	MN		
	Median (IQR)			*p* value^1^	*p* value^2^
Lipocalin-2, ng/ml	15.0 (8.1)	14.0 (5.4)	14.3 (5.5)	crude 0.47 adj. 0.81	0.99 0.92
Ferritin, µg/L	33.1 (69.2)	87.7 (92.8)	74.3 (126.2)	crude 0.18 adj. 0.39	0.20 0.20
Iron, µmol/L	14.2 (8.0)	15.5 (9.5)	14.3 (7.4)	crude 0.47 adj. 0.60	0.61 0.73
Soluble transferrin receptor, mg/L	2.9 (1.1)	2.8 (1.5)	3.1 (1.6)	crude 0.28 adj. 0.10	0.12 0.072
Transferrin, g/L	2.7 (0.4)	2.6 (0.5)	2.8 (0.5)	crude 0.88 adj. 0.67	0.32 0.83
Transferrin saturation, %	24 (13)	21 (16)	22 (12)	crude 0.92 adj. 0.50	0.37 0.78
Hemoglobin, g/L	138 (16)	146 (14)	147 (19)	crude 0.11 adj. 0.15	0.20 0.13

Values are untransformed median (IQR) values. Statistical significance tested by independent samples ANOVA, independent-samples T-test and ANCOVA after log-transformation with Bonferroni correction as applicable. Crude=unadjusted model. Adj.= model adjusted for iFGF23, cFGF23, PTH and age.

WNT1: n=17; PLS3: n=14; MN: n=34. One sample missing from lipocalin-2; one value missing from hemoglobin, and three values from other iron parameters.

^1^p value for comparison between WNT1, PLS3 and MN subjects.

^2^p value for comparison between MP (WNT1 and PLS3) and MN subjects.

WNT1, WNT1 mutation-positive subjects; PLS3, PLS3 mutation-positive subjects; MN, mutation-negative subjects; iFGF23, intact fibroblast growth factor 23; cFGF23, C-terminal fibroblast growth factor 23; PTH, parathyroid hormone.

## Results

### Subjects

The cohort comprised 17 *WNT1* mutation-positive patients (hereafter referred to as WNT1 MP) with median age 52 years (range 11–76 years; 12 females) and 14 *PLS3* mutation-positive patients (PLS3 MP) with median age 41 years (range 8–76 years; 9 females), and 34 mutation-negative control subjects (MN) with median age 36 years (range 8–77 years; 17 females). The severity of skeletal fragility differed in the mutation-positive subjects with varying number of previous fractures (range 0 to >10 fractures), vertebral compression fractures and osteoporosis medications. Treatment for osteoporosis was ongoing for three subjects at the time of study. The use of medication was similar in all study cohorts.

### Biochemical results

Clinical and biochemical characteristics of the study cohort are summarized in **Table 1**. Mean 1,25[OH]_2_D concentration tended to be higher among PLS3 MP subjects than others, however, in a pairwise comparison no significant differences were observed. The majority of subjects had normal iron status (within reference range for age) with no differences between groups; only isolated findings of supra- and subnormal values were observed (**Table 1**). More females (4/38 [11%]) had iron deficiency (ferritin below reference) than males (0/24 [0%]), however, the difference was not statistically significant (Chi-Square p=0.26). None of the children had iron deficiency, and mean ages were 46 years among iron deficient and 39 years in non-iron deficient subjects (p=0.14). Plasma levels of lipocalin-2 did not differ between WNT1 MP (median 15.0 ng/ml; range 9.4–64.7 ng/ml) or PLS3 MP (median 14.0 ng/ml; range 8.7–21.4 ng/ml) and MN (median 14.3 ng/ml; range 8.8–196.1 ng/ml) subjects (**Table 2**). Males and females had similar lipocalin-2 concentrations (*p*=0.41), also within groups of MP and MN subjects (*p*>0.13). Furthermore, adults and children had similar lipocalin-2 levels (*p*>0.19). We observed no differences in iron status markers between groups (**Table 2**).

### Association between lipocalin-2 and other biomarkers

**Figure 1:**
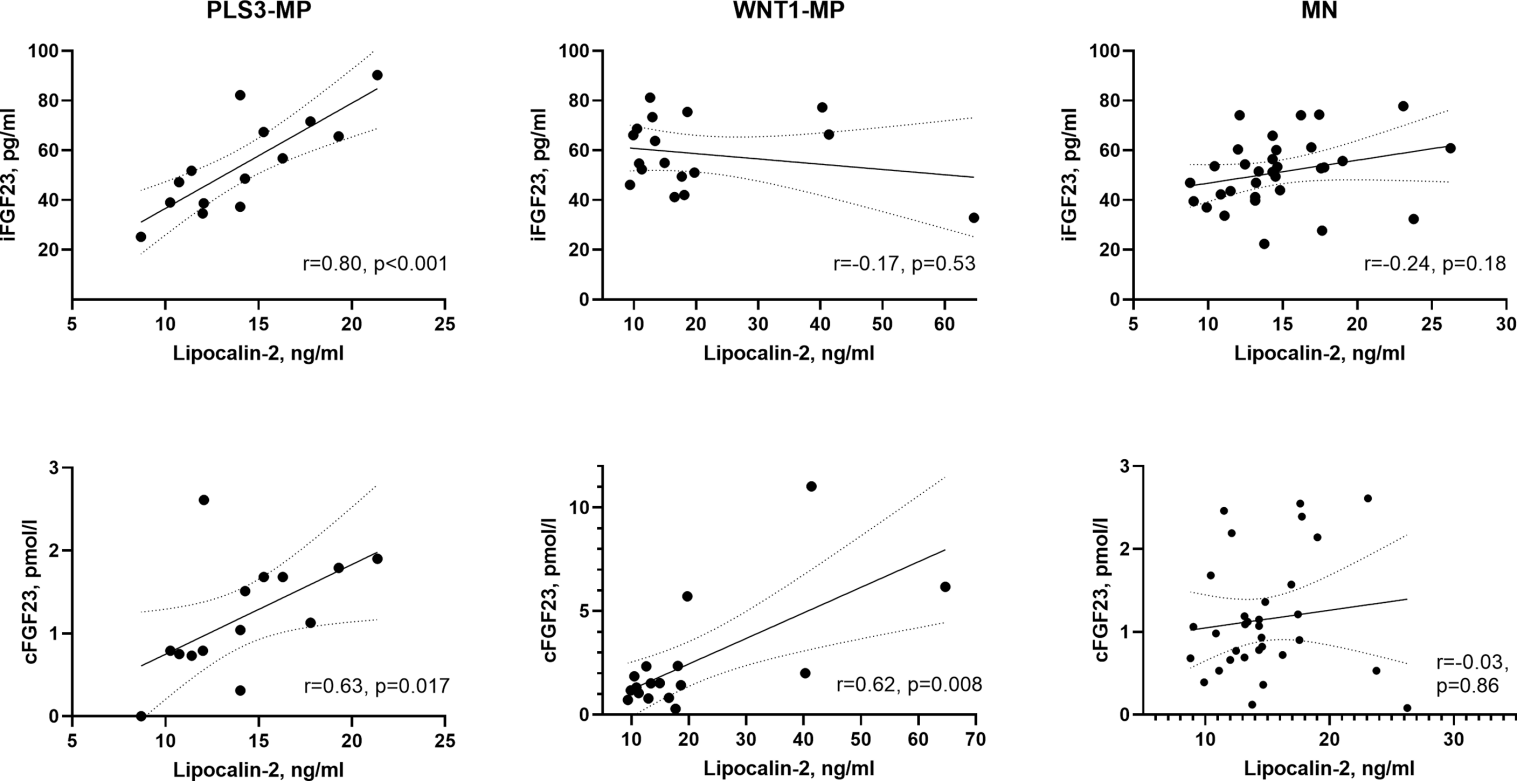
Scatter plots between lipocalin-2 and iFGF23, and lipocalin-2 and cFGF23 in PLS3, WNT1 and MN subjects separately. One outlier from MN subjects was excluded from figure. Pearson correlation coefficients (r) were calculated after log-transformation. Line represents simple linear regression with 95% confidence bands of the best-fit line. PLS3-MP, *PLS3* mutation-positive subjects; *WNT1* mutation-positive subjects; MN, mutation negative subjects; iFGF23, intact fibroblast growth factor 23; cFGF23, C-terminal fibroblast growth factor 23.

**Figure 1** illustrates crude correlations between lipocalin-2 and FGF23 in WNT1, PLS3 and MN subjects. We observed a positive correlation between lipocalin-2 levels and iFGF23 in PLS3 MP subjects (*r*=0.80, *p*<0.001) but no correlation in WNT1 MP subjects (*p*=0.53) or MN subjects (*p*=0.18). In PLS3 (*r*=0.63, *p*=0.017) and WNT1 MP (*r*=0.62, *p*=0.008) subjects, lipocalin-2 correlated with cFGF23. Lipocalin-2 did not correlate with cFGF23 in MNs subjects (*p*=0.86).

Correlations between lipocalin-2 and other biomarkers of bone metabolism and iron status are shown in **Table 3**. Lipocalin-2 correlated with CTX (*r*=0.49, *p*=0.045), soluble transferrin receptor (*r*=0.53; *p*=0.029) and transferrin (*r*=0.72, *p*=0.001), and negatively with ferritin (*r*=-0.51, *p*=0.036) and transferrin saturation (*r*=-0.52, *p*=0.034) in WNT1 MP subjects but not in PLS3 MP or MN subjects (*p* for all >0.31). Lipocalin-2 did not correlate with DKK1 in any subgroup (*p*>0.21).

**Table 3:** Correlations between lipocalin-2 and other biomarkers of bone metabolism and iron status in *WNT1* and *PLS3* mutation-positive subjects, and MN family controls.

Lipocalin-2								
	All ( n = 64 )		WNT1 ( n = 14 )		PLS3 ( n = 17 )		MN ( n = 33 )	
	r	*p*	r	*p*	r	*p*	r	*p*
cFGF23	0.28	**0.025**	0.62	**0.008**	0.63	**0.017**	-0.03	0.86
iFGF23	-0.05	0.71	-0.17	0.53	0.80	**<0.001**	-0.24	0.18
DKK1	-0.21	0.21	-0.23	0.38	-0.05	0.87	-0.08	0.65
Sclerostin	0.16	0.20	-0.01	0.98	0.40	0.16	0.18	0.31
PINP	0.22	0.08	0.50	0.050	-0.18	0.54	0.19	0.28
Phosphate	-0.03	0.79	0.05	0.85	0.36	0.20	-0.15	0.41
1,25[OH]_2_D	-0.11	0.79	0.31	0.23	-0.44	0.11	-0.17	0.34
PTH	-0.11	0.37	-0.41	0.10	-0.06	0.83	0.00	0.99
ALP	-0.06	0.62	-0.40	0.11	0.35	0.21	0.07	0.72
CTX	0.19	0.14	0.49	**0.045**	-0.03	0.91	0.05	0.79
Ionized calcium	0.05	0.69	0.15	0.56	-0.18	0.54	-0.03	0.87
Ferritin	-0.14	0.28	-0.51	**0.036**	0.05	0.87	0.03	0.85
Iron	-0.13	0.30	-0.40	0.11	-0.19	0.54	-0.01	0.96
TfR	0.06	0.65	0.53	**0.029**	-0.31	0.31	-0.08	0.66
Transferrin	0.08	0.54	0.72	**0.001**	-0.15	0.63	-0.14	0.45
Transferrin saturation	-0.14	0.27	-0.52	**0.034**	-0.15	0.64	0.03	0.86
Hemoglobin	-0.06	0.62	-0.02	0.95	0.20	0.64	-0.10	0.60

Pearson correlation coefficients (r) after log-transformation as applicable.

Two values missing from iron status markers, except for hemoglobin.

WNT1, WNT1 mutation-positive subjects; PLS3, PLS3 mutation-positive subjects; MN, mutation-negative subjects; cFGF23, C-terminal fibroblast growth factor 23; iFGF23, intact fibroblast growth factor 23; DKK1, dickkopf-1; PINP, type I procollagen intact N-terminal propeptide; 1,25[OH]_2_D, 1,25-dihydroxyvitamin D; PTH, parathyroid hormone; ALP, alkaline phosphatase; CTX, collagen type I cross-linked C-telopeptide; TfR, soluble transferrin receptor.P values <0.05 highlighted in bold.

## Discussion

The current study investigated whether lipocalin-2 could be a novel biomarker for WNT1 and PLS3 osteoporosis and explored its relationship to other parameters of bone and mineral metabolism. WNT1 and PLS3 pathogenic variants cause early-onset, severe osteoporosis with low BMD and frequent fractures. Diagnostic biomarkers for these disorders are limited and the pathogenic mechanisms still incompletely understood. We found that plasma lipocalin-2 levels did not differ between *WNT1* or *PLS3* mutation-positive subjects compared with mutation-negative healthy subjects. However, we observed a correlation between lipocalin-2 and FGF23 in *WNT1* and *PLS3* mutation-positive subjects and between lipocalin-2 and iron status in *WNT1* mutation-positive subjects, while such correlations were absent in mutation-negative subjects.

WNT1 and PLS3 are two crucial factors regulating bone metabolism and abnormalities in their signaling have been linked to severe early-onset osteoporosis (20). The pathway for WNT1 is quite well known: WNT1 signals through the canonical WNT/beta-catenin pathway by binding to a transmembrane dual co-receptor consisting of LRP5/LRP6 and seven transmembrane G-protein Frizzled (FZD), initiating an intracellular cascade resulting in increased bone formation and decreased bone resorption. DKK1 and sclerostin are two osteocyte-released factors and the main WNT1 inhibitors. The actions of PLS3 in bone are less well known: PLS3 is known to bind to and crosslink actin structures into larger fibers but the key factors it interacts with to regulate bone formation remain unknown. Despite the grave skeletal fragility in both disorders, we have previously reported that conventional metabolic bone markers are normal in both WNT1 and PLS3 patients, and hence additional novel biomarkers would be needed.

Lipocalin-2 has been shown to partake in bone homeostasis and associate with bone parameters (13–16, 18); however, the exact mechanisms in bone metabolism remain unclear. Because of increased levels of FGF23 in WNT1 patients and DKK1 in PLS3 patients (7) and the previously reported link between lipocalin-2 and bone parameters, lipocalin-2 could have a role in relation to aberrant WNT1 or PLS3 signaling.

Our results suggest that lipocalin-2 is not a specific biomarker for WNT1 and PLS3 osteoporosis, but the correlation between lipocalin-2 and FGF23 indicates an association between these markers in bone biology. FGF23 is an important osteokine and a key regulator of bone homeostasis. Bone and the bone marrow are the main sources of circulating FGF23. The production and cleavage of FGF23 is tightly regulated by bone mineral factors but also by other factors such as inflammation, iron, erythropoietin, energy levels and metabolic factors. FGF23 regulates phosphate homeostasis by increasing phosphate excretion in urine and reducing intestinal absorption thereby lowering serum phosphate levels (21). In previous studies, we observed a high expression of FGF23 in bone biopsies and increased level of serum intact and C-terminal FGF23 but normal phosphate parameters in *WNT1* patients (7, 22). This was considered to be secondary to the iron-deficient microenvironment in bone as the WNT1 patients also exhibited low bone marrow iron storage (7). We have previously reported normal levels of phosphate excretion in these patients and controls (7).

In the present study, we found a correlation between lipocalin-2 and FGF23 in *WNT1* and *PLS3* patients, suggesting a close relationship between these osteokines. This could be *via* their shared regulatory factors, such as inflammation, iron homeostasis and erythropoiesis, or by direct interaction, as has been reported previously (23). Mouse studies have recently shown that lipocalin-2 mediates the expression of *Fgf23* in osteoblasts and osteocytes in response to inflammation and in chronic kidney disease. The direct stimulation of FGF23 production in bone by lipocalin-2 is at least partly mediated by activation of cAMP signaling in bone cells. The excess lipocalin-2 in kidney disease increases bone production of FGF23 and serum FGF23 levels, which contributes to the progression of the disorder (24). Decreasing FGF23 levels by inhibition of lipocalin-2 has been suggested as a therapeutic approach to improve outcomes in chronic kidney disease (23). The patients in our study did not present with kidney disease. The kidney-bone axis is still incompletely understood, and it remains unclear how lipocalin-2 is linked to the function of the osteocytes. The correlation between FGF23 and lipocalin-2 found in our patients may be related to inflammatory responses or iron homeostasis. Iron has been associated with FGF23 in healthy children (25, 26). Lipocalin-2 regulates iron homeostasis in physiological and inflammatory conditions by transporting iron and regulating systemic, cytosolic and mucosal iron levels (27, 28). Of note, we also observed a negative correlation between lipocalin-2 and ferritin, the major iron storage protein in the body, in *WNT1* patients, which could be related to the low bone marrow iron stores in *WNT1* patients. The observed correlations between lipocalin-2 with transferrin, soluble transferrin receptor, and the degree of transferrin saturation further supports the association of lipocalin-2 to iron hemostasis and functional iron depletion.

We have previously reported elevated levels of DKK1 in *PLS3* mutation-positive subjects (7). DKK1 has been shown to correlate with lipocalin-2 in healthy adult women (16). In the present study, lipocalin-2 did not correlate with DKK1 in *PLS3* or *WNT1* patients or in mutation-negative healthy subjects. This could in part be explained by the differences in studied cohorts as our cohort consisted of both men and women from all age groups.

We recognized the limitations in our study, especially concerning the relatively small cohort size and lack of directly age- or gender-matched controls. Replication of the results in a larger cohort is limited by the rarity of WNT1 and PLS3 mutation-positive patients. Also, our study was performed in a cross-sectional setting. Furthermore, due to limited patient information on potential confounders such as body mass index or renal function we were not able not evaluate the possible influence of these parameters on lipocalin-2 levels. However, the subjects in our study were of normal weight and did not present with kidney disease or diabetes. No renal abnormality has previously been linked to WNT1 or PLS3 osteoporosis. Further, ferritin results have to be interpreted with caution since we did not systematically measure the concurrent acute inflammation status, which can elevate the circulating levels of ferritin (29). Nevertheless, the association between lipocalin-2 with transferrin, soluble transferrin receptor and transferrin saturation supports the link to iron hemostasis. Despite these recognized limitations, given the lack of prior data on lipocalin-2 in monogenic skeletal disorders or in relation to other bone biomarkers, and the rarity of affected mutation-positive subjects with monogenic skeletal disorders, such as WNT1 and PL3 osteoporosis, we consider our results valuable and unique.

In conclusion, our study demonstrates that the circulating levels of lipocalin-2 are not altered in WNT1 and PLS3 osteoporosis, and therefore cannot be used as a metabolic biomarker in the diagnosis or for monitoring these two monogenic conditions. However, our results indicate a link between lipocalin-2 and FGF23 in abnormal WNT1 or PLS3 signaling and associations between lipocalin-2 and parameters of iron status in abnormal WNT1 signaling. Further studies are required to investigate the significance of these associations, the involved biological mechanisms, and the role of lipocalin-2 in bone biology.

## Data availability statement

The original contributions presented in the study are included in the article/Supplementary Material . Further inquiries can be directed to the corresponding author.

## Ethics statement

The studies involving human participants were reviewed and approved by Research Ethics Board of Helsinki University Hospital. Written informed consent to participate in this study was provided by the participants’ legal guardian/next of kin.

## Author contributions

Conceptualization: OM, PM, RM; Data curation, formal analysis, and methodology: PL, HH-A; PM, RM; Funding acquisition: OM, PM, RM; Investigation: PL, HH-A, PM, RM; Project resources, administration and supervision: OM, PM, RM; Software and validation: PL, HH-A, PM, RM; Visualization: PL, HH-A, RM; Roles/Writing - original draft: PL, HH-A, RM; Writing - review & editing: all authors. All authors contributed to the article and approved the submitted version.

## Funding

This work was funded by University of Helsinki and Helsinki University Hospital through the Doctoral Programme in Clinical Research, the Finnish Medical Foundation, Päivikki and Sakari Sohlberg Foundation, Juho Vainio Foundation, Sigrid Juse´lius Foundation, Novo Nordisk Foundation, Folkhälsan Research Foundation, the Finnish–Norwegian Research Foundation, Orion Research Foundation, The Finnish ORL– HNS Foundation and ALF grants from Region Östergötland. The funders were not involved in the study design, collection, analysis, interpretation of data, the writing of this article or the decision to submit it for publication.

## Acknowledgments

We are grateful to all participants in this study. We thank Päivi Turunen and Mira Aronen for help with sample collection, and Diana Atanasova and Sari Linden for their excellent technical support.

## Conflict of interest

The authors declare that the research was conducted in the absence of any commercial or financial relationships that could be construed as a potential conflict of interest.

## Publisher’s note

All claims expressed in this article are solely those of the authors and do not necessarily represent those of their affiliated organizations, or those of the publisher, the editors and the reviewers. Any product that may be evaluated in this article, or claim that may be made by its manufacturer, is not guaranteed or endorsed by the publisher.
